# Modulation Effects of Eugenol on Nephrotoxicity Triggered by Silver Nanoparticles in Adult Rats

**DOI:** 10.3390/biology11121719

**Published:** 2022-11-27

**Authors:** Hanaa R. Aboelwafa, Ramadan A. Ramadan, Somaya S. Ibraheim, Hany N. Yousef

**Affiliations:** Biological and Geological Sciences Department, Faculty of Education, Ain Shams University, Cairo 11566, Egypt

**Keywords:** silver nanoparticles, eugenol, kidney, oxidative stress, histology, histopathology, immunohistochemistry

## Abstract

**Simple Summary:**

The main objective of this study is to assess how eugenol (Eug) affects AgNP-induced nephrotoxicity in rats. After 30 days of treatment with AgNPs, rats developed nephrotoxicity, which was characterized by disruptions in the serum levels of blood urea nitrogen, creatinine, uric acid, the total oxidant capacity, the total antioxidant capacity, and interfering levels of kidney injury molecule-1, superoxide dismutase, catalase, reduced glutathione, glutathione peroxidase, malondialdehyde, tumor necrosis factor-alpha (TNF-α), and interleukin-6 in kidney tissues. These biochemical alterations were accompanied by the destruction of normal renal architecture, with most renal components shedding their thickness, diameter, and quantity. Furthermore, P53, Caspase3, and TNF-α immunoreactivity were considerably elevated; however, Bcl-2 immunoreactivity was reduced. Most biochemical, histological, histomorphometrical, and immunohistochemical alterations in AgNP-treated rats were reversed by Eug. We may infer that Eug has a protective effect against AgNP-induced nephrotoxicity.

**Abstract:**

The use of silver nanoparticles (AgNPs) is expanding. This study evaluates the modulator effect of eugenol (Eug) on AgNP-induced nephrotoxicity in rats. Sixty male rats were separated into six groups: control, Eug, AgNPs low-dose, AgNPs high-dose, Eug + AgNPs low-dose, and Eug + AgNPs high-dose. After 30 days, kidney function, antioxidative and proinflammatory status, histopathological, histomorphometrical, and immunohistochemical assessments were performed. AgNPs markedly induced oxidative stress in renal tissues, characterized by increased levels of blood urea nitrogen, creatinine, uric acid, kidney injury molecule-1, the total oxidant capacity, malondialdehyde, tumor necrosis factor-alpha (TNF-α), and interleukin-6, as well as decreased levels of the total antioxidant capacity, superoxide dismutase, catalase, reduced glutathione, and glutathione peroxidase. Moreover, the normal renal architecture was destroyed, and the thickness of the renal capsules, cortex, and medulla, alongside the diameter and quantity of the normal Malpighian corpuscles and the proximal and distal convoluted tubules were decreased. Immunoreactivity for P53, caspase-3, and TNF-α reactive proteins were significantly increased; however, Bcl-2 immunoreactivity was decreased. Eug reversed most biochemical, histological, histomorphometrical, and immunohistochemical changes in AgNP-treated animals. This study demonstrated that nephrotoxicity in AgNP-treated rats was mitigated by an Eug supplementation. Eug’s antioxidant, antiapoptotic, and anti-inflammatory capabilities were the key in modulating AgNPs nephrotoxicity.

## 1. Introduction

Silver nanoparticles (AgNPs) are one of the most researched nanomaterials worldwide [1]. Generally, AgNPs are 1–100 nm nanoparticles with special physical, chemical, and biological characteristics that have various applications [2].

AgNPs have been widely used in biomedicine, food packing, water disinfection, and personal care products due to their antimicrobial properties [2,3]. Furthermore, AgNPs have been employed in various products, including surgical tools, contact lenses, urinary dilators, intrauterine devices, cannulas, pacemakers, hospital bedding disinfection, fabrics, food products, and electrical home appliances [4,5,6]. According to the estimates, there are already more than 1000 consumer items that use AgNPs, and the yearly production of AgNPs reaches over 800 tons [7].

Environmental concerns have arisen as we use nanomaterials more frequently in our daily lives. AgNPs can enter the body inadvertently or on purpose through inhalation, drinking, ingestion, skin absorption, or an intravenous injection [8]. AgNPs can translocate and accumulate in several body tissues, including the liver, kidneys, lungs, heart, and nerves, where they can impair the cellular metabolism and induce toxicity [9].

The pathways driving AgNP-induced cytotoxicity and genotoxicity are not well defined. Two probable mechanisms of the adverse effects of AgNPs are hypothesized to be the release and production of reactive oxygen species (ROS) caused by silver ions [10]. Elevated amounts of intracellular ROS result in oxidative stress, lipid peroxidation, and further cellular macromolecular degradation, which ultimately results in cell death [11].

Nanoparticles’ physiochemical properties determine how they interact with cells and, as a result, their overall potential toxicity. According to earlier studies [12,13,14], AgNPs with particle sizes ranging from 10 to 100 nm have toxic effects at concentrations ranging from 0.0082 to 1 mg kg^−1^.

Recently, various studies have highlighted the ability of phytochemicals to act as antioxidants [15]. One of the several phenolic phytochemicals found in clove, cinnamon, and basil is eugenol (Eug) (C_10_H_12_O_2_; phenylpropanoid), which is utilized as a flavoring substance in food and cosmetics [16]. In terms of the pharmacological effects, Eug has been shown to have antibacterial [17], antiviral [18], antifungal [19], anti-inflammatory [20], antioxidant and free radical scavenging [21], and anticancer [22] properties.

According to the present literature, the impact of AgNP produced by the microwave technique on the kidney of mammals and the ability of Eug to mitigate AgNP-induced nephrotoxicity has only been studied in extremely infrequent and generalized ways. Therefore, this study was designed to determine Eug’s possible modulation impact on AgNP-induced nephrotoxicity in male rats using biochemical, histological, histomorphometrical, and immunohistochemical procedures.

## 2. Materials and Methods

### 2.1. Chemicals Used

Eug (99%), silver nitrate (AgNO_3_, 99%), and gelatin were obtained from Sigma-Aldrich (St. Louis, MO, USA). The reagents and chemicals used were of a high purity and analytical grade.

### 2.2. Production of AgNPs

According to Zahran et al. [23], the microwave technique was used to create AgNPs from gelatin and AgNO_3_. For 20 min, 16.987 g of AgNO_3_ was dissolved in 100 mL of deionized distilled water. Then, the AgNPs were dissolved in 1 g gelatin soluble in deionized distilled water. On a direct hot plate, the solution was agitated for 24 h at 60 °C, and for the final 5 min, it was microwave-irradiated at 700 watts. The temperature of the solution was naturally cooled to room temperature, securely sealed, and protected.

### 2.3. Physicochemical Features of AgNPs

In our previous study [24], the AgNPs used in this investigation were characterized using different techniques. Scanning electron microscopy and transmission electron microscopy revealed that the particles were monodispersed, transparent, spherical, and highly crystalline, with a normal small size (7.77–28.4 nm), smooth surface, well distribution, and no agglomeration. Furthermore, ultraviolet-visible spectroscopy, X-ray diffraction patterns, and X-ray photoelectron spectroscopy showed that the particles had a face-centered cube structure and good crystallinity, as described in our previous study.

### 2.4. Experimental Animals

Sixty adult males *Rattus norvegicus* of a similar age (3–4 months) and weight (180–200 g) were purchased from the animal house of Theodor Bilharz Research Institute located in El-Giza, Egypt. The rats were housed in hygienic plastic containers filled with wood shavings and received a typical pellet diet for rodents in addition to unrestricted access to water at room temperature (25 ± 2 °C), with a 12 h light–dark cycle and a relative humidity of 55 ± 5%. All the rats were given a week to acclimate before the experiment. This investigation complied with the international criteria for animal laboratory treatment which oversees the use and treatment of animals locally set by the Institutional Animal Ethics Committee of Ain Shams University.

### 2.5. Experimental Design

All animals were divided into six groups (n = 10 rats for each group). For 30 days, the rats received the following treatment every day at 9 a.m.:

Control group: the healthy rats were given deionized distilled water (1 mL, intraperitoneally (i.p.)) as the vehicle for AgNPs and corn oil (1 mL, orally) as the vehicle for Eug.

Eug-treated group: the rats were orally administered with 100 mg kg^−1^ body weight of Eug suspended in 1 mL of corn oil. This dosage was determined using data from earlier rat investigations [25].

Low-dose AgNP-treated group: the animals were i.p. injected with 1 mg kg^−1^ body weight of AgNPs dissolved in 1 mL of deionized distilled water.

High-dose AgNP-treated group: the animals were i.p. injected with 2 mg kg^−1^ body weight of AgNPs dissolved in 1 mL of deionized distilled water.

The low and high doses of AgNPs used in this study were selected according to the doses used in earlier investigations [8].

Eug + low-dose AgNP-treated group: the animals were given 100 mg kg^−1^ body weight of Eug by oral gavage simultaneously with an i.p. injection of 1 mg kg^−1^ body weight of AgNPs (low dose).

Eug + high-dose AgNP-treated group: the animals were given 100 mg kg^−1^ body weight of Eug by oral gavage simultaneously with an i.p. injection of 2 mg kg^−1^ body weight of AgNPs (high dose).

### 2.6. Retrieving Samples of Sera and Tissues

After the completion of the treatment period, the animals from all groups were nightly starved and necropsied the next morning under mild anesthesia. Through a heart puncture, blood samples were collected and centrifuged for 10 min at 1500× *g* and 4 °C to obtain the sera, which were then immediately frozen at 80 °C until use. In order to conduct additional biochemical studies, the kidneys of the dissected rats were taken out, quickly rinsed with ice-cold physiological saline (0.9% NaCl), and then maintained frozen at 80 °C. To conduct the histological, histomorphometrical, and immunohistochemical evaluations, additional kidney specimens were removed and processed.

### 2.7. Preparation of Kidney Homogenates

Kidney samples were homogenized in ice-cold phosphate-buffered saline (PBS) which has a pH value of 7.4 to get a 10% solution (*w*/*v*) by an Ultra Turrax tissue homogenizer. To remove any erythrocytes and clots, the PBS was mixed with 0.16 mg/mL heparin. The formed homogenate was centrifuged at 9000× *g* and 4 °C for 15 min., and subsequently the lucid supernatant was collected and stored frozen at −80 °C for further biochemical experiments. The previously described method [26] that employed bovine serum albumin as a standard were used to determine the quantity of the kidney protein content in each sample.

### 2.8. Biochemical Assessment

#### 2.8.1. Kidney Function Biomarkers

Colorimetric assay kits tailored for kidney function biomarkers [blood urea nitrogen (BUN), creatinine, and uric acid] were purchased from EGY-CHEM for lab technology (BioMed, Hannover, Germany) and utilized simultaneously with a UV-VIS spectrophotometer (Shimadzu, Kyoto, Japan) to determine BUN, creatinine, and uric acid levels in the sera according to previously reported protocols by Tietz [27].

#### 2.8.2. Kidney Injury Molecule-1 (KIM-1)

Using an ELISA kit (Catalog Number. CSB-E08808r, Cusabio Biotech Co., Ltd., Houston, TX, USA), the levels of Kidney Injury Molecule-1 (KIM-1) in renal tissue homogenates were assessed following the experimental procedure provided in the instruction manual.

#### 2.8.3. Oxidative Stress Biomarkers in Sera

The total antioxidant capacity (TAC) and total oxidant capacity (TOC) of the sera were evaluated using colorimetric test kits made by Biomedica Medizinprodukte GmbH, Germany following the procedures previously described by Koracevic et al. [28] and Tatzber et al. [29], respectively.

#### 2.8.4. Oxidative Stress Biomarkers in Kidney Tissues

The lipid peroxidation (LPO) levels were determined using a colorimetric test kit (Biodiagnostic, Giza, Egypt) based on the generation of thiobarbituric acid reactive substances (TBARS), and represented as the quantity of malondialdehyde (MDA) creation in accordance with Buege and Aust [30].

Using commercially available colorimetric kits (Biodiagnostic, Giza, Egypt) and a UV-vis spectrophotometer (Shimadzu, Kyoto, Japan), the antioxidant activities of superoxide dismutase (SOD), catalase (CAT), reduced glutathione (GSH), and glutathione peroxidase (GPx) in kidney tissues were determined (Shimadzu, Kyoto, Japan). To measure the efficiency of SOD, the procedure previously described by Nishikimi [31] was used. H_2_O_2_ consumption was used as a proxy for the CAT activity, as indicated by Aebi [32], whilst the GSH and GPx activities were measured using the methodologies previously illustrated by Beutler et al. [33] and Paglia and Valentine [34], respectively.

#### 2.8.5. Proinflammatory Markers in Kidney Tissues

In kidney tissues, two pro-inflammatory cytokines, tumor necrosis factor-alpha (TNF-α), and interleukin 6 (IL-6) were measured. Following the manufacturer’s instructions, the TNF-α levels were determined utilizing commercially available enzyme-linked immunosorbent assay (ELISA) kits (catalogue number: CSB-E11987r, Cusabio Biotech Co., Ltd.) and the concentration of IL-6 was determined using the Rat IL-6 Quantikine ELISA Kit (R & D Systems, Inc., Minneapolis, MN, USA).

### 2.9. Histological Preparation

Both the control and treated animal kidney samples were split into small pieces and immediately fixed for 24 h in Bouin’s fixative. They then underwent the standard paraffin sectioning technique as described earlier [35]. Ehrlich’s hematoxylin and eosin (H&E) were used to stain the obtained 4–6 μm thick paraffin sections that were then dehydrated using graded concentrations of ethyl alcohol, cleared using xylene, mounted using DPX, inspected using a compound light microscope (Olympus CX 31), and finally photographed using a Panasonic CD-220 camera connected with the microscope.

### 2.10. Histomorphometrical Estimation

Six randomly selected fields from the H&E-stained kidney sections of all the experimental groups were histomorphometrically examined in the Department of Oral and Dental Pathology, Dental Medicine Faculty, Al-Azhar University using a computerized image analysis system (Leica QWin, 500 image analysis software, Leica Microsystems, Wetzlar Germany). For each selected kidney section, the thickness of the fibrous renal capsule, renal cortex, and renal medulla were measured. Additionally, the diameter of the Malpighian corpuscles and proximal and distal convoluted tubules were estimated. Additionally, the number of normal and damaged Malpighian corpuscles and proximal and distal convoluted tubules were recorded.

### 2.11. Immunohistochemical Preparation

Using the standard avidin–biotin complex (ABC) protocol [36], the responsive proteins Bcl-2, P53, caspase-3 (Cas3), and TNF-α were immunohistochemically estimated in the kidney tissues from all the experimental animal groups. The sectors of renal tissue that were 4–6 μm thick, buffered formalin-fixed, and paraffin-embedded were dewaxed, rehydrated, and washed in PBS for 10 min. Hydrogen peroxide at a concentration of 3% was used to prevent endogenous peroxidase activity. The slides were then conveniently diluted with the primary antibodies shown in Table 1 for 1–2 h at room temperature, then were kept at 4 °C in a refrigerator nightly. After that, they were rinsed in PBS, exposed to biotinylated goat anti-polyvalent for 10 min, and subsequently exposed to ABC for 1 h. The slices were washed in PBS before being treated for 7–9 min in diaminobenzidine tetrahydrochloride (pH 7.2) with 10 mL of H_2_O_2_. Subsequently, the sections were dehydrated, cleared, counterstained with Mayer’s hematoxylin, coated with cover slips, examined, and captured. For each parameter, negative control slides with no primary antibodies were provided. All the reagents and antibodies were used in accordance with the manufacturer’s instructions and suggestions.

### 2.12. Image Analysis

**Figure 1:**
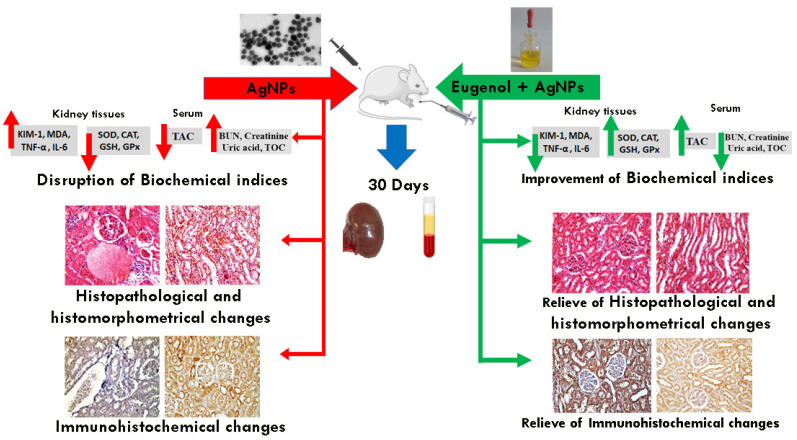
Schematic representation of the study design and main results.

To quantify the immunoreactivity of Bcl-2, P53, Cas3, and TNF-α reactive proteins, image analysis was used to determine the proportion of immuno-positive cells to the total number of cells evaluated for each parameter [37]. If the cytoplasm or membranous coloration was brown, the cells were considered to be positive. Six high-power fields (X200) were chosen for each parameter and recorded in each slide using a standard measuring frame with an area of 11434.9 mm^2^. The image analyzer was initially programmed to automatically convert the image analyzer program’s measurement units (pixels) to actual micrometer units. The mean percentages of the immunoreactive area for all the samples in each group were computed for statistical analysis using a Leica QWin 500 (Leica Microsystems, Wetzlar, Germany) computational image analysis system in the Department of Oral and Dental Pathology, Faculty of Girls’ Dental Medicine, Al-Azhar University. A graphic description of the approach used, and the key conclusions, is shown in Figure 1.

### 2.13. Statistical Analysis

The obtained biochemical, histomorphometrical, and immunohistochemical data were tabulated and statistically analyzed. All the experimental animal sets’ values for six samples per group were displayed as the mean standard error of mean (SEM). A one-way analysis of variance (ANOVA) was used to quantify the statistical differences between the groups of rats, and then the IBM SPSS Statistics for Windows, version 22 (IBM Corp., Armonk, NY, USA) was utilized for the Tukey post hoc test. When a *p*-value was less than 0.05, a statistical significance was considered.

## 3. Results

### 3.1. Biochemical Analysis

To evaluate kidney injury, the kidney function indicators (BUN, creatinine, and uric acid), and the oxidant/antioxidant biomarkers (TOC & TAC) in the sera, in addition to KIM-1, the oxidative stress indices (SOD, GSH, CAT, GPx, and MDA) and inflammation markers (TNF-α and IL-6) in the kidney tissues were assessed.

Table 2 shows the serum levels of BUN, creatinine, and uric acid in the rats of all the groups. The results depicted no significant differences (*p* ≥ 0.05) in the estimated kidney function biomarkers between the rats treated with Eug alone and the corresponding control animals. Meanwhile, both the low- and high-dose AgNP-treated rats exhibited a marked elevation (*p* ≤ 0.05) in BUN (130.45% and 246.94%), creatinine (189.68% and 857.14%), and uric acid (29.21% and 67.15%), respectively, compared with those in the control group. In the rats which were given either a low or high dosage of AgNPs, an Eug supplementation modulated these estimated parameters compared with those in the animals who were given AgNPs alone, although the results were still significantly different (*p* ≤ 0.05) from the control values.

**Figure 2:**
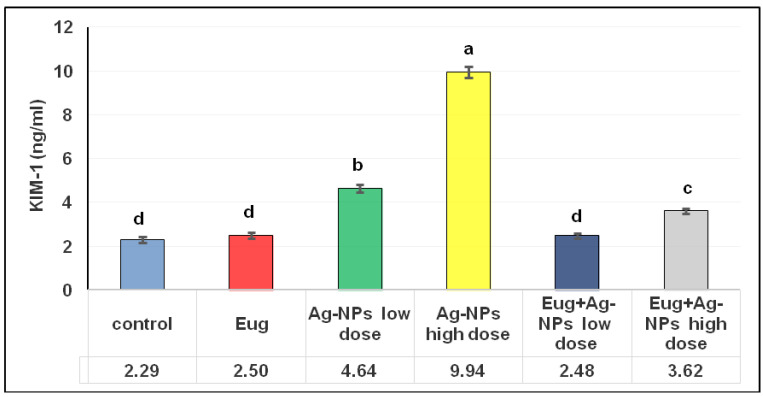
Levels of kidney injury molecule-1 (KIM-1) in renal tissues of the control and treated animal groups. The data are presented as mean ± SEM (*n* = 6). Values in the same row that are followed by different superscript letters differ significantly at 5% (*p* ≤ 0.05) level of significance according to ANOVA test and the TUKEY test. Eug, eugenol; AgNPs, silver nanoparticles.

As illustrated in Figure 2, an administration of Eug alone did not have any significant effects on the KIM-1 levels (*p* ≥ 0.05) compared with the control data. Meanwhile, low- and high-dose AgNP-treated rats showed a marked increase (*p* ≤ 0.05) in KIM-1 levels (102.18% and 333.50%, respectively) compared with the control group. The administration of low-dose AgNPs alongside Eug to the rats resulted in KIM-1 values that were substantially similar to the KIM-1 values in the control group (2.48 ± 0.12 vs. 2.29 ± 0.13; *p* ≥ 0.05); however, the concomitant administration of Eug to rats intoxicated with a high dose of AgNPs decreased this parameter compared with those in the rats treated with AgNPs alone, but this value remained significantly different (*p* ≤ 0.05) from the control values.

The serum levels of TAC and TOC in the rats in each group are shown in Table 3. A marked increase (*p* ≤ 0.05) in the TAC levels (16.07%) was observed in the Eug-treated group compared with those in the control group. In contrast, a non-significant decline (*p* ≥ 0.05) and a significant reduction (*p* ≤ 0.05) in the TAC levels in the low-dose (−9.19%) and high-dose (−55.01%) AgNP-treated rats were recorded compared with those in the control group. The levels of TAC in AgNP-treated rats, in both low and high doses, were increased by an Eug supplementation compared with those in the rats treated with AgNPs alone; however, TAC levels in high-dose AgNP-treated rats were significantly different (*p* ≤ 0.05) from the control values. The administration of Eug alone had no significant impact (*p* ≥ 0.05) on the levels of TOC compared with the control group. However, a remarkable increase (*p* ≤ 0.05) in the TOC levels were observed in the low-dose (49.90%) and high-dose (130.98%) AgNP-treated rats compared with those in the control group. When the rats were given a low dose of AgNPs along with Eug, the levels of TOC were very similar to those in the control animals (355.5 ± 12.76 vs. 351.5 ± 10.44; *p* ≥ 0.05), although when the rats were given a high dose of AgNPs along with Eug, this parameter declined compared with that in the animals treated with AgNPs alone; however, this value remained significantly different (*p* ≤ 0.05) from the control values.

To track the oxidative stress status, the levels of MDA, SOD, CAT, GSH, and GPx in the kidney tissues of the control and treated animal groups were evaluated. Table 4 shows that the administration of Eug alone had no noticeable (*p* ≥ 0.05) impact on these oxidative stress indicators. Both the low- and high-dose AgNP-treated rats experienced oxidative stress, which was demonstrated as a significant increase (*p* ≤ 0.05) in the MDA levels (36.31% and 308.92%, respectively) and a sharp decrease (*p* ≤ 0.05) in the levels of SOD (−27.20% and −58.22%, respectively), CAT (−36.24% and −63.11%, respectively), GSH (−45.12% and −61.91%, respectively), and GPx (−32.75% and −60.51%, respectively) compared with those in the control group. The evaluated oxidative stress biomarkers were significantly modulated (*p* ≤ 0.05) in the Eug + low-dose AgNP-treated and Eug + high-dose AgNP-treated groups compared with those in the animals exposed to low- and high-dose AgNPs alone; however, most parameters remained statistically different (*p* ≤ 0.05) from the control values.

The TNF-α and IL-6 levels in renal tissues were examined in all groups, and the within-group values of these parameters were compared (Table 5). The rats treated with Eug alone showed non-significant (*p* ≥ 0.05) alterations in TNF-α and IL-6 levels compared with the control group. In contrast, the rats treated with either a low or high dose of AgNPs showed significantly elevated levels (*p* ≤ 0.05) of TNF-α (101.89% and 311.14%, respectively) and IL-6 (56.01% and 148.93%, respectively) compared with the control animals. The administration of Eug with AgNPs (low or high dose) significantly (*p* ≤ 0.05) attenuated these alterations compared with those in animals exposed to low- and high-dose AgNPs alone and restored the levels to the control values.

### 3.2. Histological Results

**Figure 3:**
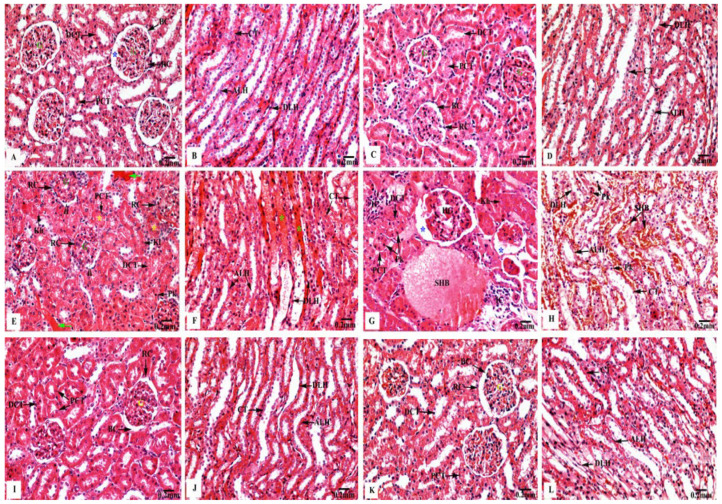
Kidney sections of control and treated animal groups stained with H&E displaying (**A**,**B**) orderly renal architecture with outstandingly organized renal corpuscles (RC) forming of Bowman’s capsules (BC) enclosing normal Bowman’s spaces (blue asterisks) surrounding the glomeruli (G), in addition to the proximal (PCT) and distal (DCT) convoluted tubules in the cortical zone, as well as normal descending (DLH) and ascending (ALH) limps of the loops of Henle, and collecting tubules (CT) in medullary area of control rats; (**C**,**D**) regular cortical and medullary renal structure in Eug-treated rats; (**E**,**F**) hazardous effects on renal cortical portion represented by altered renal corpuscles (RC) which have decreased Bowman’s spaces (arrowheads) with expansion of their glomeruli (G), and focal tubular necrosis where the proximal (PCT) and distal (DCT) convoluted tubules are filled with hyaline casts or cellular debris (yellow asterisks), as well as their epithelia showed nuclear pyknosis (Pk), karyorrhexis (Kh), or karyolysis (Kl). Severely deteriorated inter-tubular blood vessels with hemorrhagic blood masses (green arrows). Additionally, renal medulla appeared with conspicuous damage of the lining cells of the descending (DLH) and ascending (ALH) limbs of the loops of Henle and collecting tubules (CT), as well as extravasated hemolyzed blood (green asterisks) were seen in AgNPs low-dose-treated rats; (**G**,**H**) severely deteriorated renal cortex with malformed renal corpuscles showing hypertrophied glomeruli (HG) and increased Bowman’s spaces (blue asterisks), as well as the proximal (PCT) and distal (DCT) convoluted tubules appeared with pyknotic (Pk) or karyorrhexed (Kh) nuclei. The inter-tubular spaces were filled with infiltrated inflammatory cells (IC) and stagnant hemolyzed blood (SHB). Additionally, deteriorated renal medulla with stagnant hemolyzed blood (SHB) in the inter-tubular spaces and marked coagulative necrosis of the lining cells of the descending (DLH) and ascending (ALH) limbs of the loops of Henle, and the collecting tubules (CT) represented with nuclear pyknosis (Pk) were seen in AgNPs high-dose-treated rats; (**I**,**J**) remarkable improvement in the renal tissues’ histological structure, with regular cortical and medullary regions were recorded in Eug + AgNPs low-dose-treated rats; (**K**,**L**) restoration of the kidney structure was noticed in the renal cortex and medulla in Eug + AgNPs high-dose-treated rats.

The kidney sections of the control (Figure 3A,B) and Eug-treated (Figure 3C,D) rats exhibited a normal histological architecture, with a well-organized fibrous capsule, renal cortex, and renal medulla. In the renal cortex, numerous Malpighian corpuscles were observed, consisting of a double membrane Bowman’s capsule, and enclosing Bowman’s spaces and a tuft of blood capillaries or the glomeruli. Additionally, the proximal convoluted tubules appeared with round or oval outlines and narrow lumens, and their lining layer consisted of simple tall cuboidal epithelium having distinct brush borders in their free narrow apices, markedly basophilic cytoplasm, and centrally located nuclei; the distal convoluted tubules also had larger and more clearly defined lumens with cuboidal epithelial lining cells as more nuclei per cross-section were observed in the renal cortex (Figure 3A,C). The renal medullary region enclosed the descending and ascending limbs of the loops of Henle and collecting tubules. The descending limb had very thin walls and comprised flattened squamous epithelium, bounding a rather wide lumen; they had a homogenously eosinophilic cytoplasm, and their nuclei were bulging into the internal lumens. Meanwhile, the ascending limb had abruptly thick walls and appeared lined by low cuboidal epithelial cells, enclosing a narrow lumen, and their cytoplasm was also generally eosinophilic; their cell boundaries were ill-defined but had centrally situated conspicuous nuclei, and exhibited deep basophilia. The collecting tubules were lined by a simple cuboidal epithelium, which became increasingly tall distally; they had a poorly stained cytoplasm, embodying large oval nuclei located in the middle position of these cells, as illustrated in Figure 3B,D.

Meanwhile, the renal tissues of low-dose AgNP-treated rats revealed marked harmful responses, as seen in Figure 3E,F. The renal cortex appeared with deteriorated Malpighian corpuscles, showing a decreased Bowman’s spaces with an expansion or mesangial hypercellularity of the glomeruli and focal tubular necrosis where the proximal and distal convoluted tubules were filled with hyaline casts or cellular debris; moreover, their lining epithelial cells showed nuclear pyknosis, karyorrhexis, or karyolysis. Severely deteriorated inter-tubular blood vessels with hemorrhagic blood masses were also observed. The renal medulla appeared with conspicuous damage of the lining epithelia of the descending and ascending limbs of the loops of Henle and the collecting tubules. Furthermore, extravasated hemolyzed blood was observed.

Moreover, as shown in Figure 3G,H, the renal tissues of high-dose AgNP-treated rats exhibited significant pathological changes, where the renal corpuscles appeared with hypertrophied glomeruli and increased the Bowman’s spaces; additionally, the proximal and distal convoluted tubules appeared with pyknotic or karyorrhexed nuclei. The deteriorated renal medulla revealed marked coagulative necrosis of the lining cells of the descending and ascending limbs of the loops of Henle and collecting tubules with nuclear pyknosis were recorded. Additionally, the inter-tubular spaces were filled with infiltrated inflammatory cells and stagnant hemolyzed blood.

In contrast, the rats treated with Eug + low-dose AgNPs showed an obvious improvement in their renal architecture, including intact renal corpuscles having normal Bowman’s capsules and glomeruli. In addition, they had normal proximal and distal convoluted tubules, where the supplementation of Eug partly returned their lining cells to their normal arrangement. Moreover, the descending and ascending limbs of the loops of Henle and the collecting tubules had clear signs of regeneration of their lining epithelial cells, with their lumens appearing clear (Figure 3I,J). Similarly, Eug + high-dose AgNP-treated rats displayed a relatively well-organized renal architecture, including all the components of the renal capsule, cortex, and medulla (Figure 3K,L).

### 3.3. Histomorphometrical Results

**Figure 4:**
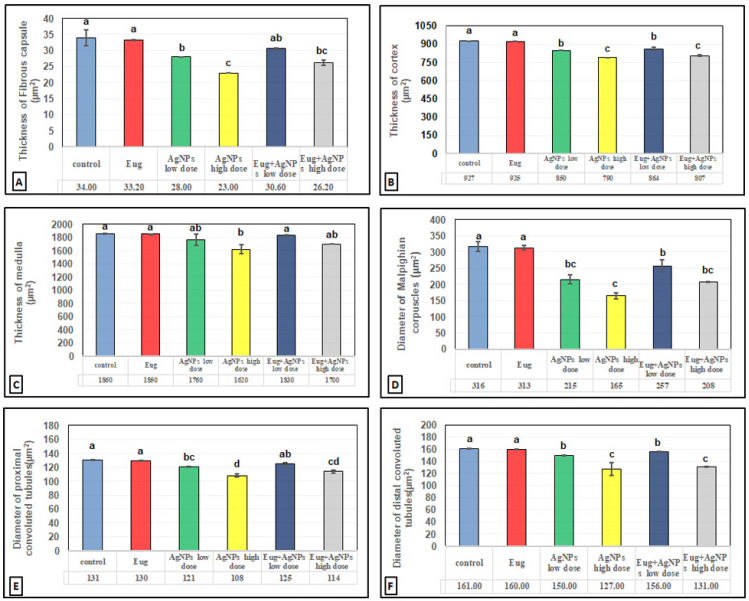
Histomorphometrical evaluation of renal tissue components in the control and treated animal groups showing (**A**) the thickness of fibrous renal capsules (μm^2^), (**B**) the thickness of renal cortex (μm^2^), (**C**) the thickness of renal medulla (μm^2^), (**D**) the diameter of Malpighian corpuscles (μm^2^), (**E**) the diameter of proximal convoluted tubules (μm^2^), and (**F**) the diameter of distal convoluted tubules (μm^2^). Each column represents mean ± SEM, *n* = 6. Means with different superscript letters differ significantly at 5% (*p* ≤ 0.05) level of significance according to ANOVA test and the TUKEY test. Eug, eugenol; AgNPs, silver nanoparticles.

**Figure 5:**
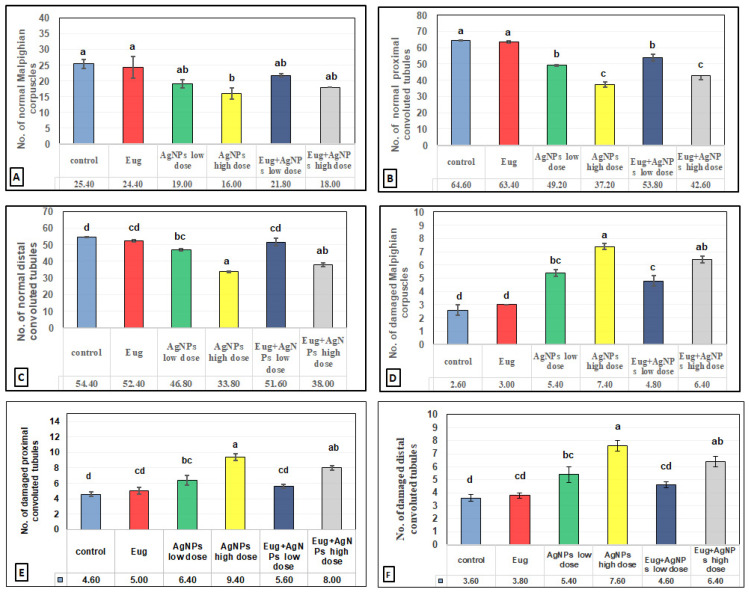
Histomorphometrical evaluation of renal tissue components in the control and treated animal groups showing the number of (**A**) normal Malpighian corpuscles, (**B**) normal proximal convoluted tubules, (**C**) normal distal convoluted tubules, (**D**) damaged Malpighian corpuscles, (**E**) damaged proximal convoluted tubules, and (**F**) damaged distal convoluted tubules. Each column represents mean ± SEM, *n* = 6. Means with different superscript letters differ significantly at 5% (*p* ≤ 0.05) level of significance according to ANOVA test and the TUKEY test. Eug, eugenol; AgNPs, silver nanoparticles.

The toxic impacts of AgNPs and the protective effects of Eug on renal tissues were demonstrated in several renal histomorphometrical estimations, as seen in Figure 4A–F. The AgNP treatment, either of a low or high dose, significantly decreased (*p* ≤ 0.01) the thickness of the fibrous renal capsules (−17.65% and −32.35%), renal cortex (−8.31% and 14.78%), renal medulla (−5.38% and −12.90%), and the diameter of the Malpighian corpuscles (−31.96% and −47.78%), proximal convoluted tubules (−7.63% and −17.56%), and distal convoluted tubules (−6.83% and −21.12%), respectively. Additionally, a high significant decrease (*p* ≤ 0.01) in the numbers of normal Malpighian corpuscles (−25.20% and −37.01%), normal proximal convoluted tubules (−23.84% and −42.41%), and normal distal convoluted tubules (−13.97% and −37.87%) paralleled with a significant increase (*p* < 0.05) in the number of damaged Malpighian corpuscles (+107.69% and +184.62%), damaged proximal convoluted tubules (+39.13% and +104.35%), and damaged distal convoluted tubules (+50% and +111.11%), respectively, which were recorded from the kidney sections of the rats treated with low- and high-dose AgNPs compared with the values in the control animals (Figure 5A–F).

In contrast, a co-treatment with Eug in the rats treated with either low- or high-dose AgNPs markedly modulated all the histomorphometrical parameters compared with those in animals intoxicated with low- and high-dose AgNPs, whereas most parameters were significantly different (*p* ≤ 0.05) from the recorded control values. Meanwhile, the administration of Eug alone had a non-significant (*p* > 0.05) effect on the measured renal histomorphometrical parameters compared with those in the control group, as observed in Figure 4 and Figure 5.

### 3.4. Immunohistochemical Results

#### 3.4.1. Bcl-2 Immunoreactivity

**Figure 6:**
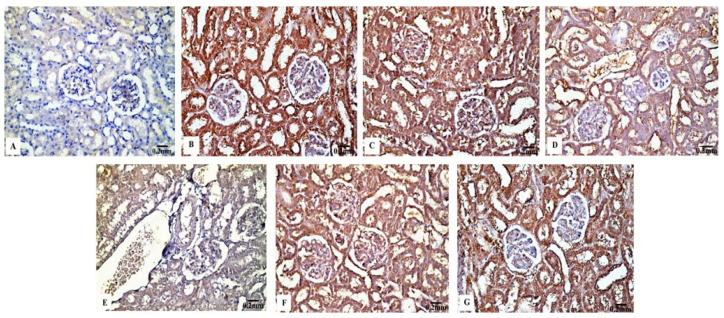
Immunohistochemical evaluation of Bcl-2 expression in renal tissues of the control and treated animal groups showing (**A**) no staining in the negative control, (**B**) an intense immunostainability in the control group, (**C**) a strong immunoreaction in the Eug-treated group, (**D**) a moderate immunoreaction in the low-dose AgNPs-treated group, (**E**) a weak immunostainability in the high-dose AgNPs-treated group, (**F**) a moderate immunostaining in Eug + AgNPs low-dose-treated group; (**G**) a modest immunostainability in the group treated with Eug + AgNPs at a high dosage.

In contrast to the renal tissues obtained from the control rats (Figure 6B) and Eug-treated-rats (Figure 6C), which displayed robust Bcl-2 immunoreactivity, the kidney sections obtained from the rats treated with low-dose AgNPs showed a moderate Bcl-2 immunoreactivity (Figure 6D), whereas the kidney sections of high-dose AgNP-treated rats revealed a poor Bcl-2 immunoreactivity (Figure 6E). In the renal tissues of rats from the low-dose (Figure 6F) and high-dose (Figure 6G) AgNP groups, the immunoexpression of Bcl-2 was upregulated when Eug was concurrently administered with AgNPs. Negative immunoreactivity was observed in the negative control sample (Figure 6A). As seen in Figure 10A, no significant difference (*p* > 0.05) in the area percentage of Bcl-2 immunoexpression was observed between Eug-treated and control rats. However, both the low- and high-dose AgNP-treated groups showed a substantial decrease (*p* ≤ 0.05) in the area percentage of the Bcl-2 immunoexpression, which was considerably regulated in rats treated with Eug.

#### 3.4.2. P53 Immunoreactivity

**Figure 7:**
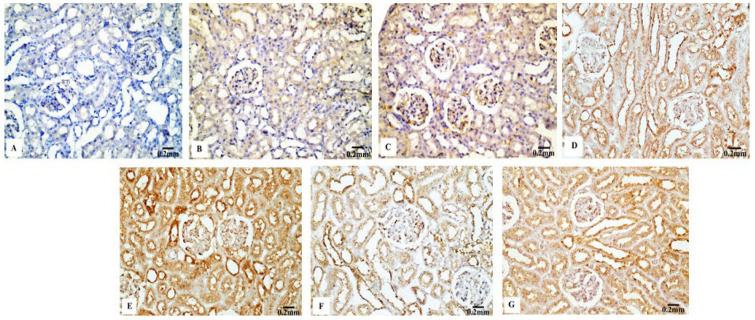
Immunohistochemical evaluation of P53 expression in renal tissues of the control and treated animals groups showing (**A**) a negative immunostaining in the negative control, (**B**) a weak immunostainability in the control group, (**C**) a weak immunostainability in the Eug-treated group, (**D**) a moderate immunoreaction in low-dose AgNPs-treated group, (**E**) a strong immunostainability in the AgNPs high-dose-treated group, (**F**) a weak affinity for P53 in the Eug + ANPs low-dose-treated group, and (**G**) a modest immunostainability in the group treated with Eug + AgNPs at a high dosage.

The P53 immunoreactivity in the kidney tissues of normal (Figure 7B) and Eug-treated (Figure 7C) rats appeared weak; however, a moderate P53 immunoreactivity was observed in low-dose AgNP-treated rats (Figure 7D). Moreover, the rats treated with a high dose of AgNPs showed an intensely positive immunoreactivity to P53 (Figure 7E). Meanwhile, the renal sections of rats treated with Eug+ low-dose AgNPs displayed a weak P53 immunoreactivity (Figure 7F), and those of the rats treated with Eug + high-dose AgNPs showed a moderate response to P53 (Figure 7G). In the negative control samples, no staining was observed (Figure 7A). Statistically, no considerable difference (*p* > 0.05) in the area percentage of P53 immunoexpression between the Eug-treated and control groups was detected. Meanwhile, P53 immunoexpression was significantly increased (*p* ≤ 0.05) in both the low- and high-dose AgNP-treated groups. In contrast to the low- and high-dose AgNP-treated groups, the concomitant administration of Eug modulated the area percentage of P53 immunoexpression, however, the area percentage of P53 immunoexpression still showed a considerable increase (*p* ≤ 0.05) compared with that in the control rats (Figure 10B).

#### 3.4.3. Cas3 Immunoreactivity

**Figure 8:**
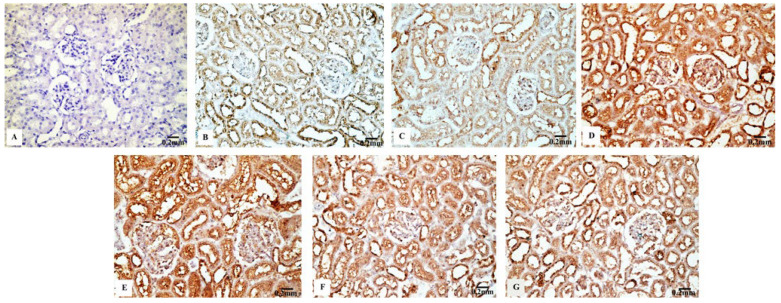
Immunohistochemical analysis of Caspase-3 (Cas3) expression in renal tissues of the control and treated animal groups showing (**A**) no stainability in the negative control, (**B**) a weak immunostaining in the control group, (**C**) a weak immunoreaction in the Eug-treated groups, (**D**) a high positive immunostainability in the low-dose AgNPs-treated group, (**E**) a strong immunoreaction in the AgNPs high-dose-treated group, and (**F**,**G**) a modest immunostainability in both groups treated with Eug paralleled with either the low or high dose of AgNPs.

The renal tissues from the control (Figure 8B) and Eug-treated rats (Figure 8C) showed a weak Cas3 immunostainability. In contrast, the kidney sections from rats treated with low-dose AgNPs displayed a high positive Cas3 immunostaining (Figure 8D), and rats treated with high-dose AgNPs exhibited a strong Cas3 immunostainability in their renal tissues (Figure 8E). A moderate Cas3 immunoreactivity was observed in the groups treated with AgNPs at low and high doses when Eug was also supplied (Figure 8F,G). The negative control sample (Figure 8A) showed a negative Cas3 immunostainability. Figure 10C shows that while low- and high-dose AgNP-treated rats elicited a significant elevation (*p* ≤ 0.05) in the area percentage of Cas3 immunoexpression compared with the control rats, Eug-treated rats did not demonstrate a considerable increase (*p* > 0.05) in the area percentage of a Cas3 immunoexpression. In contrast to the kidney sections of rats treated with low- or high-dose AgNPs, the concurrent administration of Eug- to AgNP-treated rats modified the area percentage of the Cas3 immunoexpression but, nevertheless, it showed a substantial elevation (*p* ≤ 0.05) compared with that in the control group.

#### 3.4.4. TNF-α Immunoreactivity

**Figure 9:**
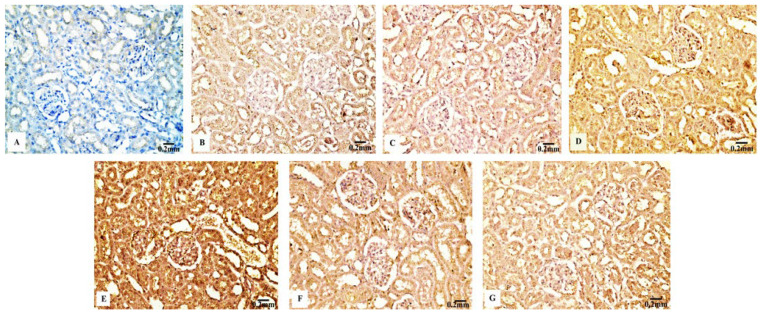
Immunohistochemical analysis of TNF-α expression in renal tissues the control and treated animal groups showing (**A**) a negative immunoreactivity in negative control, (**B**) a mild immunoreaction in the control group, (**C**) a week immunoreaction in the Eug-treated group, (**D**) an intense immunostainability in low-dose AgNPs-treated group, (**E**) a strong immunoreaction in the AgNPs high-dose-treated group, (**F**) a modest immunostainability in the Eug + AgNPs low-dose-treated group, and (**G**) a moderate immunostaining in the group treated with Eug + AgNPs at a high dosage.

**Figure 10:**
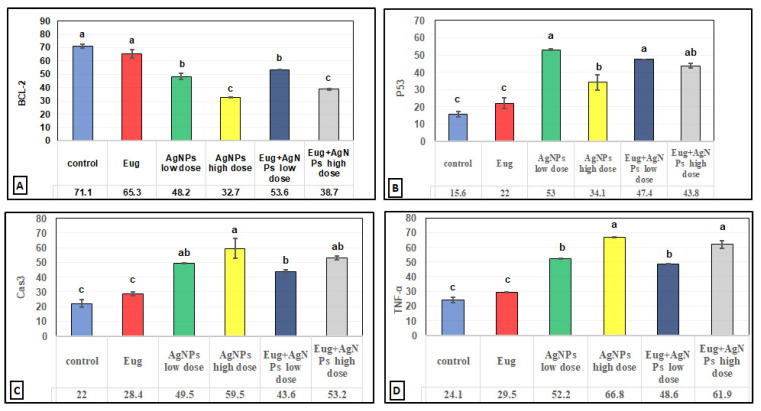
Immunohistochemical image inspection of the area percentage of the immunoexpression of the reactive proteins (**A**) Bcl-2, (**B**) P53, (**C**) Cas3, and (**D**) TNF-α in the renal tissues of the control and treated animal groups. Each column represents mean ± SEM, *n* = 6. Means with different superscript letters differ significantly at 5% (*p* ≤ 0.05) level of significance according to ANOVA test and the TUKEY test. Eug: eugenol; AgNPs: silver nanoparticles.

The kidney sections of the control rats (Figure 9B) and Eug-treated animals (Figure 9C) displayed a mild TNF-α immunohistochemical reactivity; however, the renal tissues of the rats treated with AgNPs at low and high doses (Figure 9D,E) displayed a strong positive TNF-α immunostainability. In contrast to the renal tissues of rats treated with low- or high-dose AgNPs alone, the renal tissues of rats co-administered with Eug and low-dose AgNPs (Figure 9F) or Eug and high-dose AgNPs (Figure 9G) showed a modest TNF-α immunoreactivity. Figure 9A shows a negative control sample with a negative TNF-α immunoreaction. Figure 10D shows that the area percentage of the TNF-α immunoexpression in kidney tissues did not differ significantly (*p* > 0.05) between the Eug-treated and control rats. Compared with the control group, the renal tissues of the rats treated with low- and high-dose AgNPs showed a substantial increase (*p* ≤ 0.05) in the area percentage of a TNF-α immunoexpression. However, when Eug was administered to rats receiving AgNPs at low or high doses, an TNF-α immunoexpression was modulated compared with that in rats receiving AgNPs only.

## 4. Discussion

The kidneys are highly vulnerable to the harmful influences of chemicals and medications because they are actively responsible for filtering and concentrating various substances and chemicals that may accumulate to high dangerous concentrations, which frequently result in the formation of reactive metabolites that are now regarded as key players in the pathogenesis of a renal injury [38,39]. The kidney is a unique organ for NP targeting because of its inherent ability to quickly remove particles with a diameter < 10 nm [40]. Meanwhile, a few prior investigations have examined the hazardous effects of AgNPs on the kidneys [41].

The findings of this investigation indicate AgNPs’ direct toxicity to the kidneys. The most accurate indicators of a healthy kidney function are the serum levels of BUN, creatinine, and uric acid [42]. The results of this study revealed that rats exposed to the applied dosages of AgNPs had a compromised kidney function compared with the matching control rats, as indicated by higher levels of the assessed renal function indices. The animals treated with AgNPs suffered from nephrotoxicity and experienced a severe renal failure [43]. These results may be explained by the possibility that AgNP-induced oxidative stress promotes the generation of many vasoactive mediators, which can impair the renal function by causing renal vasoconstriction or by lowering the glomerular capillary ultra-filtration coefficient, which consequently decreases the glomerular filtration rate [44]. Note that an oral administration of Eug to AgNP-treated rats restored the serum levels of BUN, creatinine, and uric acid, suggesting that Eug offers a defense against AgNP-induced nephrotoxicity. These findings are consistent with the literature, demonstrating Eug’s protective effect on gentamicin-induced nephrotoxicity [45].

The current results showed elevated levels of KIM-1 in the kidney tissues of rats treated with AgNPs. KIM-1 is a great biomarker of kidney damage and a predictor of histological alterations in the proximal tubules in response to various pathophysiological circumstances or toxicants [46]. Our results agreed with the findings of Elkhateeb et al. [47] who also found that rats exposed to copper oxide NPs had higher levels of KIM-1 in their urine and kidneys. KIM-1 is a protein which is expressed in the proximal tubule apical membrane in response to injury and is not typically present. The histological changes observed in the proximal convoluted tubules in this investigation provided evidence that AgNPs caused renal dysfunction and damage. In an earlier study, Gherkhbolagh et al. [48] examined the impact of AgNPs on rats’ kidneys and found necrosis, inflammatory changes, and further histological abnormalities. When Eug and AgNPs were administered concurrently, the KIM-1 levels in the kidneys were preserved. The moderating effects of Eug on the renal tubule epithelial structure are supported by our results, which agree with those reported in the literature [45,49].

In this study, increased TOC levels and decreased TAC levels in the sera, along with higher levels of lipid peroxidation end-product (MDA), lower levels of GSH, and the decreased efficacy of CAT, SOD, and GPx, indicated enhanced oxidative stress in the kidney tissues of rats treated with AgNPs. Li et al. [50] claimed that NPs harmed cells by generating ROS, including superoxide anion, H_2_O_2_, hydroxyl radical species, and nitric oxide. ROS may interact with macromolecules inside the cells, severely harming biological components [51]. In line with our findings, other investigations have demonstrated that AgNPs generate oxidative stress in kidney tissues [41,44].

Surprisingly, animals simultaneously administered Eug and AgNPs displayed an improvement in all the kidney oxidative stress biomarkers which were examined. Eug has been demonstrated to decrease lipid peroxidation by serving as a chain-breaking antioxidant and increasing the effectiveness of antioxidant enzymes [25,52]. Because of the presence of a phenolic hydroxyl group in its composition, which provides electrons to restrain free radicals, Eug exhibits antioxidant capabilities. Additionally, it prevents H_2_O_2_ from oxidizing Fe^2+^ in the Fenton reaction, causing the release of hydroxyl radicals that initiate the lipid peroxidation process [53].

In this study, increased levels of TNF-α and IL-6 in the renal tissues of AgNP-intoxicated animals served as a confirmation of the inflammatory condition. Numerous investigations have shown that proinflammatory cytokines are produced excessively in response to renal damage [54,55]. The excessive release of ROS, which activates the NF-κB signaling pathway and causes the synthesis of inflammatory cytokines, is blamed for the increase in proinflammatory cytokines [56].

The protein complex NF-κB attaches to an inhibitor and stays dormant in the cytoplasm. The release of inhibitory molecules is induced by several factors, such as ROS, TNF-α, and IL-8, which consequently translocates NF-κB into the nucleus [57]. NF-κB stimulates the transcription and expression of several genes, including those encoding inflammatory cytokines, inside the nucleus, thus fostering inflammation, oxidative stress, and apoptosis [58]. Additionally, pattern-recognition receptors build multiprotein complexes known as inflammasomes, which induce inflammation after pathogenic microorganisms and danger signals are found in the cytosol of host cells [59]. AgNPs activate the NF-κB transcriptional and inflammasome pathways, signaling their involvement in the molecular mechanism underlying AgNPs’ proinflammatory actions [60]. The overproduction of the examined inflammatory cytokines in the kidney was significantly reduced in rats treated with Eug simultaneously with either low- or high-dose AgNPs. One of the immunomodulatory and anti-inflammatory actions of Eug is the repression of the NF-κB pathway [61].

The present results revealed that AgNPs caused considerable histological and histomorphometrical alterations in the renal tissues of treated rats, which were observed in both the renal cortex and medulla. The most blatant signs of a renal deterioration were the shrinkage of the renal corpuscles, the congestion of the glomerular capillaries, tubular necrosis and degeneration, inter-tubular bleeding, and the infiltration of inflammatory cells into the peritubular and perivascular regions. Additionally, a significant reduction in the thickness of the renal capsules, cortex, and medulla, besides a marked decrease in the diameter of the Malpighian corpuscles, the proximal and distal convoluted tubules were observed. Additionally, a considerable reduction in the numbers of healthy Malpighian corpuscles and proximal and distal convoluted tubules and an elevation in the quantity of damaged Malpighian corpuscles and proximal and distal convoluted tubules were recorded. These histological findings observed after the AgNP intoxication could be explained based on the previously proposed hypothesis by Gibson and Skett [62], who reported that the mixed function oxidase system and prostaglandin endoperoxide synthase, two enzyme systems that can metabolically activate safe drugs into toxic metabolites that connect to essential cellular macromolecules and ultimately cause necrosis of the kidney tissues, are present in significant amounts in the renal tissues. Furthermore, Fogo et al. [63] highlighted that several therapeutic and diagnostic medications may contribute to tubular necrosis, which is a dose-dependent lesion, with practically all nephrons being affected and tubular cell death typically limited to the proximal tubules. According to Wang et al. [64], 20 nm NPs were riskier than 50 nm NPs because of how the renal filtration system handles different particle sizes.

The results of this study showed that after receiving AgNPs, the renal cortex was more negatively impacted than the medulla. This may have been caused in part by the unequal distribution of these particles within the kidney tissue, where approximately 90% of the renal blood flow enters the renal cortex via the circulation. Therefore, the concentration of NPs that have entered the bloodstream and gone to the cortex was relatively higher than that going to the medulla. This observation is consistent with earlier reports [65].

The histopathological findings of the lining tubular epithelial cells of the rats treated with AgNPs revealed damage including vacuolization, cloudy swelling, severe necrosis, pyknotic nuclei, and degenerative alterations, along with the desquamation of degenerated cells and shedding in the lumen of the tubules. Consistent with our findings, Abdelhalim and Jarrar [65] found cellular necrosis in the proximal tubules of gold nanoparticle (GNPs)-treated rats. Additionally, several distal tubules and collecting ducts showed signs of oxidative stress. This portion of the nephron, as previously indicated by Epstein [66], is likewise less oxygenated than the proximal area, making it more sensitive to oxidative stress caused by AgNPs.

Pandey and Srivastava [67] reported that necrosis may be an indication of the effect of AgNPs, causing swelling, lysing, and dissolving of the renal cell nuclei. Hyaline deposits were also observed in the renal tubules of the kidney tissues obtained from AgNP-administered rats, which could also be a symptom of renal injury caused by a disruption in the protein metabolism [68].

Cytoplasmic vacuolation was observed in the lining epithelia of some malformed renal tubules of AgNP-treated rats. The anomaly in renal cell membrane function that results in a substantial input of water and Na+ ions could be the origin of such cytoplasmic vacuolation. Furthermore, as previously discussed by Schrand et al. [69], the imbalance in fluid equilibrium caused by these minuscule AgNPs, which increased intracellular water, may be related to tubular degeneration. These changes may also be followed by the leakage of lysosomal hydrolytic enzymes, which can cause cytoplasmic degradation and macromolecular crowding [70].

The findings of this study further showed that some proximal and distal convoluted tubules of rats exposed to AgNPs had exfoliated epithelial cells, which indicated that AgNPs affected the adhesion of the renal cells and caused the breakdown of cell–cell junctions, in addition to the fact that oxidative stress is a critical factor in producing cell–cell separation [71]. Additionally, Pannu and Nadim [72] and Perazella [73] reported that the proximal convoluted tubules are injured due to their function in concentrating and reabsorbing glomerular filtrates. Additionally, renal tubular cells have a fast metabolic rate that uses a lot of energy and are in a very hypoxic environment, which raises the risk of hypoxic injury to the cells [74]. Furthermore, the kidneys of AgNP-treated rats occasionally displayed dilatation of the inter-tubular blood capillaries which may result from the lowering of the renal tissue vascular resistance caused by AgNPs, as previously reported [65].

One of the most evident anomalies observed following the AgNPs’ administration was a cellular infiltration. This phenomenon was first documented by Bianchi et al. [75], who related the proliferation of inflammatory cells to the harmful effects of the drugs. Additionally, according to Silva [76] and Markowitz and Perazella [77], an interstitial infiltration of the inflammatory cells signifies an allergic hypersensitivity response. Furthermore, inflammatory cells found in the renal tissues of rats treated with AgNPs indicate that these particles could interact with the proteins and enzymes of the renal interstitial tissues, interfering with the antioxidant defense mechanism and generating ROS, simulating an inflammatory response [78]. Additionally, Yen et al. [79] found that smaller NPs triggered higher immune responses than bigger ones. These inflammatory symptoms matched up with the immunohistochemistry findings, which showed a significant dose-dependent increase in the immunoexpression of the proinflammatory cytokine TNF-α in AgNP-treated groups compared with those in the control group.

Particle size significantly influences immunoreactivity because it impacts the differential complement protein deposition in NPs. Recent studies have shown how crucial the complement system is for the uptake and clearance of NPs and the regulation of the immune system’s proinflammatory response [80]. The immunohistochemical results in this study demonstrated that apoptosis is associated with AgNP-mediated cell death, which was supported by a considerable decrease in Bcl-2 immunoreactivity and an increase in P53 and Cas-3 immunoreactivities after the administration of low and high doses of AgNPs. The process of intrinsic mitochondria-mediated cell death is inhibited by Bcl-2, an antiapoptotic protein that is overexpressed in numerous malignancies. Bcl-2 does this by blocking mitochondrial membrane permeabilization, which results in the release of proapoptotic chemicals [81]. Furthermore, the family of the Bcl-2 gene regulates the intrinsic apoptotic pathway’s caspase activation, which is caused by intracellular damages, such as DNA damage [82].

P53 activation controls the production of other apoptosis-related proteins and is associated with the triggering of apoptosis [83]. Banu et al. [84] postulated that the biogenic metal NP-induced death of cancer cells was caused by promoting apoptosis and changing the expression of apoptosis-associated genes, such as Bcl-2 and P53. DNA damage is caused by the apoptotic marker Cas-3, which can be activated by intrinsic and extrinsic apoptotic mechanisms [85]. According to Sulaiman et al. [86], these alterations can be related to the accumulation of AgNPs in the tissues or to the impact of AgNPs on the mitochondrial function, which lowers the cell viability [87].

Additionally, TNF-α is involved in cell–cell communication, the proinflammatory response, and numerous inflammatory and autoimmune disorders. TNF-α is largely produced by macrophages in inflammatory tissues and contributes to tumor growth, angiogenesis, and wound healing [88]. TNF-α was shown to be overexpressed in the renal tissues of rats treated with AgNPs.

There are several pathways that can cause intracellular AgNPs nephrotoxicity, but an excessive ROS generation is the major one. AgNPs have unique properties that can pass through cell membranes and other biological barriers, causing cellular deterioration or apoptosis [89]. ROS can induce two different mechanisms of cell death, necrosis, and apoptosis. Additionally, since caspases are thought to be in charge of apoptosis, ROS activates them. Given that a biochemical decrease in GPx, CAT, and SOD is the principal cause of oxidative stress-induced cell death, such apoptotic effects may be the result of the intracellular oxidative stress brought on by AgNPs in the current study. Inflammation is one of the oxidative stress pathways that has been linked to the pathophysiology of chronic kidney disease. It has been demonstrated that excessive ROS act as mediator signaling molecules, triggering the production of inflammatory cytokines like IL-6 and TNF-α [90]. The increased expression of TNF-α and IL-6 following an exposure to AgNP in our work supports the relationship between proinflammatory cytokines and ROS and raises the possibility that excessive ROS generation contributes to AgNP-induced nephrotoxicity which is also confirmed by the current histopathological and immunohistochemical results.

When Eug was administered with AgNPs, the amplitude of the histological and histomorphometrical alterations in the renal tissues of rats treated with AgNPs at low or high doses were reduced. Additionally, animals exposed to Eug parallel with AgNPs showed the upregulation of their immunoexpression of Bcl-2, P53, Cas-3, and TNF-α reactive proteins. These outcomes agree with the previously published studies [45,91,92].

According to a recent study by Yousef et al. [24], AgNPs significantly induced oxidative stress in hepatic tissues, as evidenced by increased levels of lactate dehydrogenase, TOC, MDA, TNF-α, and IL-6, which were correlated with a significant reduction in the total TAC, SOD, CAT, GSH, and GPx levels. These abnormalities were observed in tandem with histological changes represented by the destruction of the normal liver structure, a sharp decline in Bcl-2 immunoreactivity, and a noticeable elevation in P53, Cas-3, and TNF-α immunoreactivities. Additionally, the authors reported that an Eug supplementation in AgNP-treated rats restored most of the aforementioned liver-related alterations.

## 5. Conclusions

The biochemical, histological, histomorphometrical, and immunohistochemical findings proved that rats exposed to AgNPs experience oxidative stress and inflammation, which leads to nephrotoxicity. Due to Eug’s antioxidant, antiapoptotic, and anti-inflammatory capabilities, our results also provide new light on the modulation impact of an Eug supplementation against AgNP-induced renal toxicity. Finally, we advocate the use of Eug as a protective agent along with AgNPs to reduce nephrotoxicity.

## Figures and Tables

**Table 1:** The immunohistochemical utilized antibodies.

Antibody	Bcl-2	P53	Cas3	TNF-α
Code	MA5-11757	MA5-12557	MA5-11516	MA5-23720
Clone	100/D5	DO-7	3CSP01 (7.1.44)	28,401
Antigen retrieval	PBS, pH 7.4 with 0.2% BSA	PBS, pH 7.4	PBS, pH 7.4 with 0.2% BSA	PBS with 5% trehalose
Dilution	1:50	1:100–1:200	1:50–1:100	8–25 μg/mL
Sources	Mouse/IgG, kappa	Mouse/IgG2, kappa	Mouse/IgG2a	Mouse/IgG1
Supplier	Thermo Fisher Scientific USA	Thermo Fisher Scientific	Thermo Fisher Scientific	Thermo Fisher Scientific

**Table 2:** Kidney function biomarkers: blood urea nitrogen (BUN), creatinine, and uric acid in the control and treated animal groups.

Parameters	Animal Groups					
	Control	Eug	AgNPs Low Dose	AgNPs High Dose	Eug + AgNPs Low Dose	Eug + AgNPs High Dose
BUN (mg/dL)	28.08 ± 1.77 ^e^	29.22 ± 2.17 ^e^	64.72 ± 2.78 ^b^	97.43 ± 3.36 ^a^	40.57 ± 2.03 ^d^	53.87 ± 1.81 ^c^
Creatinine (mg/dL)	0.21 ± 0.01 ^e^	0.25 ± 0.01 ^e^	0.61 ± 0.05 ^c^	2.01 ± 0.07 ^a^	0.45 ± 0.01 ^d^	0.98 ± 0.04 ^b^
Uric Acid (mg/dL)	3.02 ± 0.14 ^c^	3.12 ± 0.12 ^c^	3.9 ± 0.2 ^b^	5.05 ± 0.4 ^a^	3.27 ± 0.1 ^b,c^	3.98 ± 0.39 ^b^

The data are presented as mean ± SEM (*n* = 6). Values in the same row that are followed by different superscript letters differ significantly at 5% (*p* ≤ 0.05) level of significance according to ANOVA test and the TUKEY test. Eug, eugenol; AgNPs, silver nanoparticles.

**Table 3:** Total antioxidant capacity (TAC) and total oxidant capacity (TOC) in sera of the control and treated animal groups.

Parameters	Animal Groups					
	Control	Eug	AgNPs Low Dose	AgNPs High Dose	Eug + AgNPs Low Dose	Eug + AgNPs High Dose
TAC (mM/L)	30.12 ± 1.44 ^b,c^	34.96 ± 1.37 ^a^	27.35 ± 1.09 ^c^	13.55 ± 0.62 ^d^	35.76 ± 0.9 ^a^	30.92 ± 1.58 ^b^
TOC (μmol/L)	351.5 ± 10.44 ^d^	372.87 ± 9.9 ^d^	526.9 ± 15.73 ^c^	811.89 ± 27.12 ^a^	355.5 ± 12.76 ^d^	594.78 ± 13.85 ^b^

The data are presented as mean ± SEM (*n* = 6). Values in the same row that are followed by different superscript letters differ significantly at 5% (*p* ≤ 0.05) level of significance according to ANOVA test and the TUKEY test. Eug, eugenol; AgNPs, silver nanoparticles.

**Table 4:** Oxidative stress biomarkers: malondialdehyde (MDA), superoxide dismutase (SOD), catalase (CAT), reduced glutathione (GSH), and glutathione peroxidase (GPx) in renal tissues of the control and treated animal groups.

Parameters	Animal Groups					
	Control	Eug	AgNPs Low Dose	AgNPs High Dose	Eug + AgNPs Low Dose	Eug + AgNPs High Dose
MDA (nmol/g·tissue)	16.52 ± 0.70 ^d^	17.11 ± 0.48 ^d^	36.31 ± 2.33 ^b^	67.53 ± 1.91 ^a^	18.64 ± 1.22 ^d^	28.30 ± 1.30 ^c^
SOD (U/g·tissue)	11.90 ± 1.40 ^a^	11.69 ± 0.92 ^a^	8.66 ± 0.47 ^b^	4.97 ± 0.33 ^c^	12.80 ± 0.61 ^a^	10.79 ± 0.76 ^a,b^
CAT (U/g·tissue)	93.63 ± 2.41 ^a^	77.61 ± 15.60 ^a,b^	59.70 ± 6.69 ^b,c^	34.54 ± 2.06 ^d^	70.58 ± 2.41 ^b,c^	55.48 ± 3.11 ^c^
GSH (mmol/g·tissue)	29.90 ± 2.37 ^a^	28.80 ± 1.69 ^a,b^	16.41 ± 0.69 ^c^	11.39 ± 0.58 ^d^	32.85 ± 1.42 ^a^	25.30 ± 1.60 ^b^
GPx (U/g·tissue)	112.75 ± 3.36 ^a^	110.72 ± 3.68 ^a^	75.82 ± 4.34 ^b^	44.52 ± 3.56 ^c^	104.00 ± 2.73 ^a^	84.39 ± 2.33 ^b^

The data are presented as mean ± SEM (*n* = 6). Values in the same row that are followed by different superscript letters differ significantly at 5% (*p* ≤ 0.05) level of significance according to ANOVA test and the TUKEY test. Eug, eugenol; AgNPs, silver nanoparticles.

**Table 5:** Inflammation indicators: interleukin 6 (IL-6) and tumor necrosis factor alpha (TNF-α) in renal tissues of control and treated animal groups.

Parameters	Animal Groups					
	Control	Eug	AgNPs Low Dose	AgNPs High Dose	Eug + AgNPs Low Dose	Eug + AgNPs High Dose
TNF-α (pg/mL)	27.38 ± 1.67 ^e^	28.83 ± 1.56 ^d,e^	55.28 ± 1.88 ^c^	112.58 ± 4.73 ^a^	35.23 ± 1.79 ^d^	73.57 ± 2.29 ^b^
IL-6 (pg/mL)	55.05 ± 2.92 ^d^	56.93 ± 2.53 ^d^	85.88 ± 1.98 ^b^	137.03 ± 4.56 ^a^	60.38 ± 1.94 ^d^	72.40 ± 1.95 ^c^

The data are presented as mean ± SEM (*n* = 6). Values in the same row that are followed by different superscript letters differ significantly at 5% (*p* ≤ 0.05) level of significance according to ANOVA test and the TUKEY test. Eug, eugenol; AgNPs, silver nanoparticles.

## Data Availability

The data provided in this research are accessible from the corresponding author upon request.
